# Electrical Signals in Prayer Plants (Marantaceae)? Insights into the Trigger Mechanism of the Explosive Style Movement

**DOI:** 10.1371/journal.pone.0126411

**Published:** 2015-05-21

**Authors:** Markus Jerominek, Regine Claßen-Bockhoff

**Affiliations:** Institut für Spezielle Botanik und Botanischer Garten, Johannes Gutenberg-Universität, Mainz, Germany; University of Western Sydney, AUSTRALIA

## Abstract

The explosive pollination mechanism of the prayer plants (Marantaceae) is unique among plants. After a tactile stimulus by a pollinator, the style curls up rapidly and mediates pollen exchange. It is still under discussion whether this explosive movement is released electrophysiologically, i.e. by a change in the membrane potential (as in Venus flytrap), or purely mechanically. In the present study, electrophysiological experiments are conducted to clarify the mechanism. Artificial release experiments (chemical and electrical) and electrophysiological measurements were conducted with two phylogenetically distant species, *Goeppertia bachemiana* (E. Morren) Borchs. & S. Suárez and *Donax canniformis* (G. Forst.) K. Schum. Electric responses recorded after style release by extracellular measurements are characterised as variation potentials due to their long repolarization phase and lack of self-perpetuation. In both species, chemical and electric stimulations do not release the style movement. It is concluded that the style movement in Marantaceae is released mechanically by relieving the tissue pressure. Accordingly, the variation potential is an effect of the movement and not its cause. The study exemplarily shows that fast movements in plants are not necessarily initiated by electric changes of the membrane as known from the Venus flytrap.

## Introduction

Fast movements in plants have fascinated natural scientists for many decades. Underlying processes concerning perception of the stimulus and signal transduction were already addressed by Darwin (letters to Burdon-Sanders 15^th^ Aug. & 19^th^ Sep. 1873). He expected an electric signal mediating between stimulus and response in insectivorous plants (*Drosera* L. and *Dionaea muscipula* Ellis). Burdon-Sanders [1] tested this hypothesis for *D*. *muscipula* and measured electrical signals in plants for the first time. Such signals were characterised as action potentials by Stuhlman and Darden [2].

Examples for comparable fast plant movements are known from *Biophytum sensitivum* (L.) DC. (Oxalidaceae) and *Neptunia oleracea* Lour. (Fabaceae) [3], *Berberis vulgaris* L. (Berberidaceae) and *Sparrmannia africana* L.f. (Tiliaceae) [4], *Mimosa pudica* L. (Fabaceae) [5], *Incarvillea grandiflora* Poir. (Bignoniaceae) [6], and *Aldrovanda vesiculosa* L. (Droseraceae) [7]. For all these species electric changes were measured during the movement but interpreted differently. In *Dionaea muscipula*, the action potential appears ca. 1 s before begin of the trap movement. Bernstein [8] therefore argued that the action potential is obviously the cause of the water shift and the corresponding movement. This assumption was approved by Volkov et al. [9] who could initiate the movement experimentally by an electric stimulation of the motor cells in *D*. *muscipula*. In contrast, Bünning [4] found that the potential changes in *Berberis vulgaris* and *Sparrmannia africana* were rather the effect of the filament movement. He observed that the electric changes started not simultaneously with the change of permeability of the membrane but correlate with the water shift and the corresponding movement.

**Fig 1:**
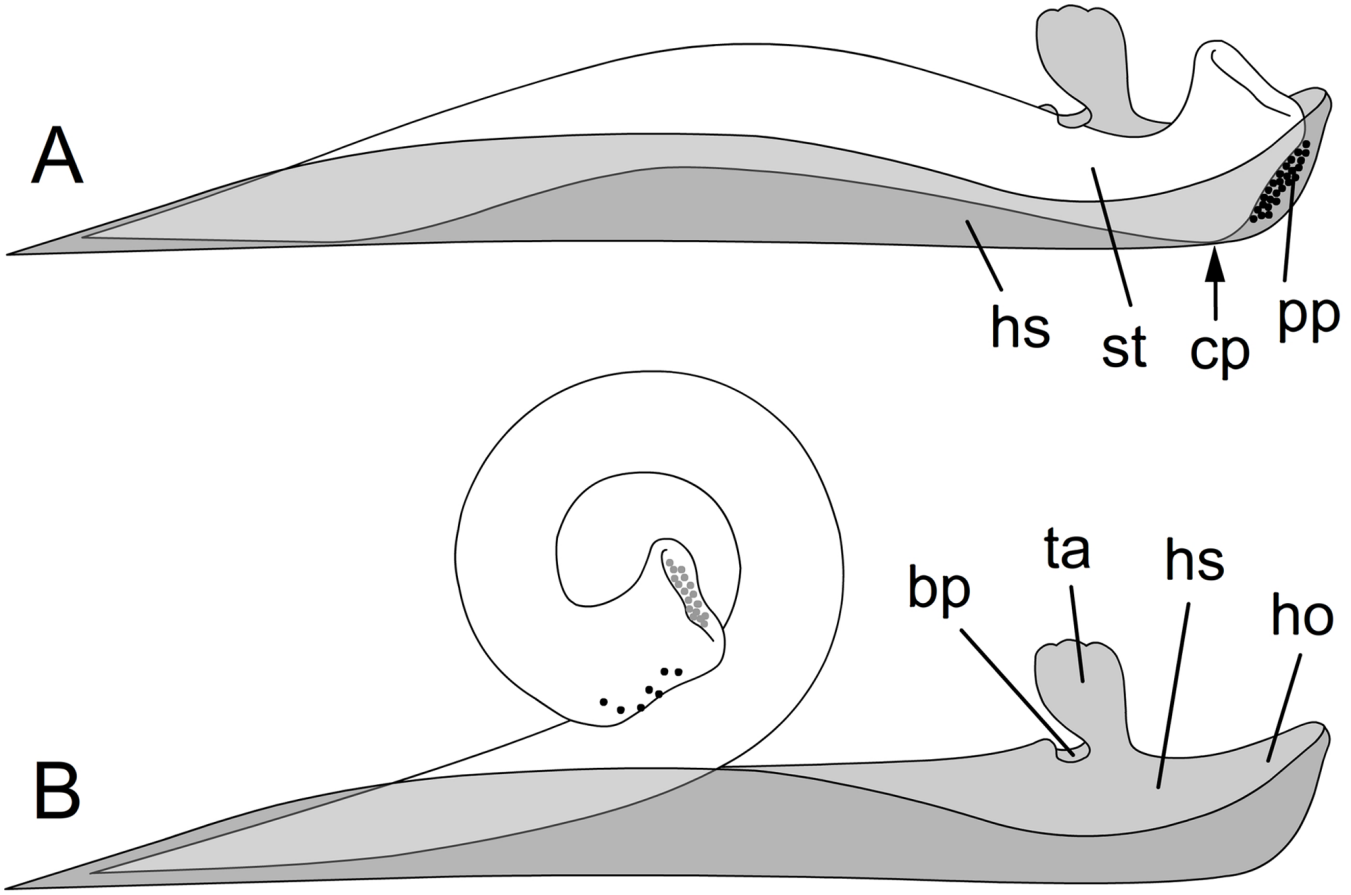
Schematic illustration of the style movement in Marantaceae. In the unreleased state (A) the style (white, st) is enveloped by the hooded staminode (grey, hs). After deflecting the trigger appendage (ta) and lifting the basal plate (bp) the style is released and curls up (B). cp, contact point; ho, hood; pp, pollen plate.

The pollination mechanism in Marantaceae mediated by an explosive style movement (Fig 1) represents a further example of rapid nastic response to tactile stimuli. Since all species of the family share this unique mechanism, Marantaceae offer a model system to study the underlying processes on a broad taxonomic scale.

**Fig 2:**
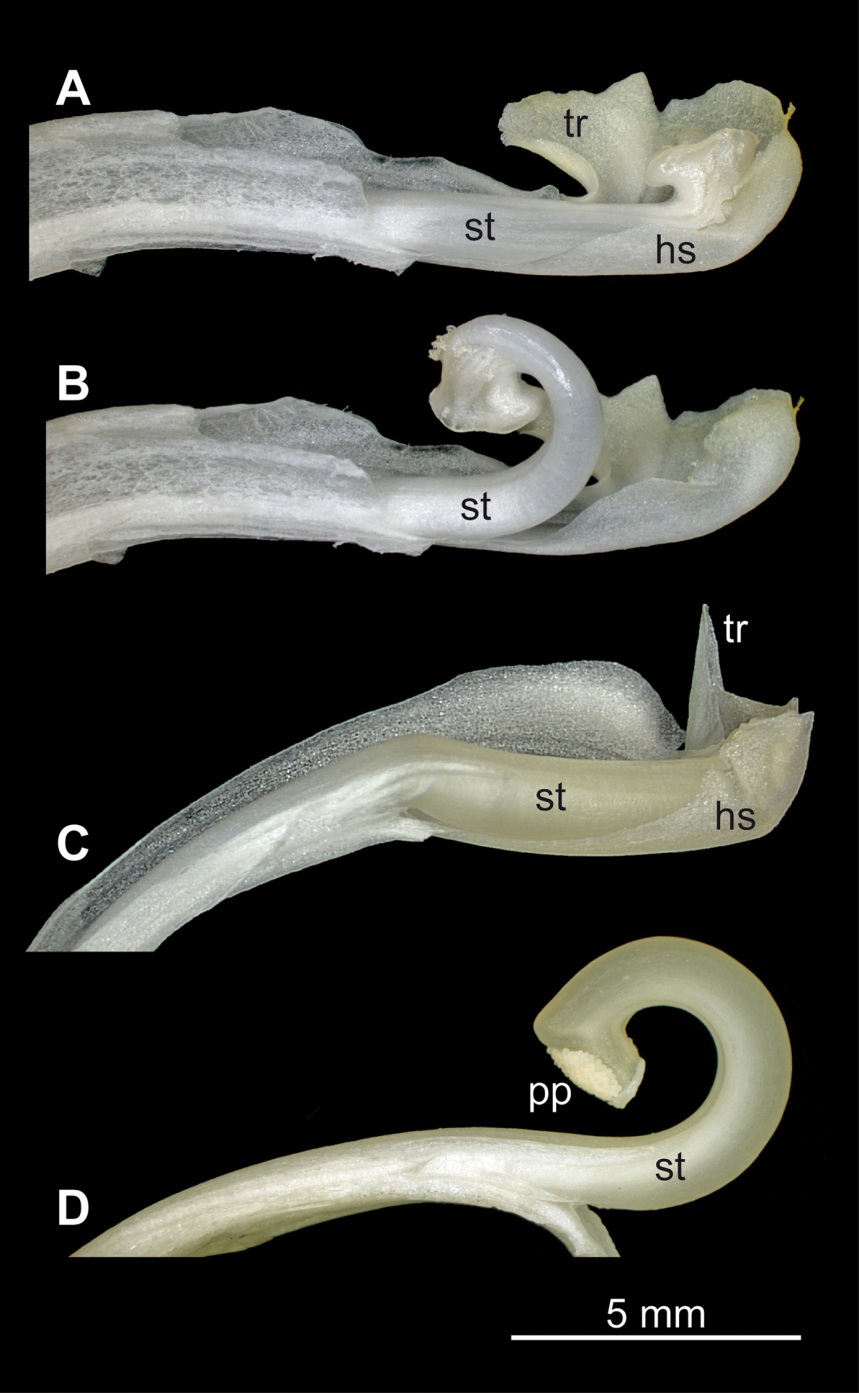
The functional unit composed of style and hooded staminode. (A, B) *Donax canniformis*, (C, D) *Goeppertia bachemiana*. (A, C) before and (B, D) after the explosive style movement. The units differ in hooded staminode (hs), style (st), trigger appendage (ta); and pollen plate (pp).

Marantaceae have an extremely modified flower morphology [10]. The inner androecial whorl includes only a single half-fertile (monothecous) anther and two sterile structures, the fleshy (callosum) and the hooded staminode (cucullatum) (Fig 1, hs). The latter forms a distal hood (ho) with a lateral folded lobe, the basal plate (bp) that merges directly into the trigger appendage (ta). In bud stage, style and anther lay tightly packed in the hooded staminode. The growing style presses the pollen out of the pollen sacs and onto the pollen plate (pp) at the head of the style [11,12]. By keeping the still growing style in the hooded staminode, mechanical tension between both organs is set up [13,14]. In many species, this is reflected by the backward bending of the style (Fig 2C). To release the style movement, the pollinator has to deflect the trigger appendage. The mechanical tension stored in the style is set free, and the style irreversibly curls up (Fig 1B). Style release can also be artificially induced by removing the hood from the style head. Thereby, the contact point (pressure point, Fig 1, cp) between the two organs is separated relieving tension [12,15].

To realise such an explosive movement without tissue rupture, the style shows a peculiar functional tissue [16]. The three vascular bundles are arranged around the style channel which is not located in the centre but at the upper half of the style. The remaining tissue is a parenchyma composed of elongated cells (like in *Mimosa pudica* [17]) which are arranged in longitudinal rows similar to the ones of *Phaseolus coccineus* L. [18]. It is rich in intracellular spaces facilitating the enormous bending. Altogether, the tissue has typical features of a motor tissue [19].

The style movement in Marantaceae was originally described as an exclusively mechanical process [20,21]. In contrast, Kunze [22] concluded from his experiments that style tension was not hold by the hooded staminode. He succeeded in inducing a movement by stinging the style under the basal plate and in removing the distal part of the hood without releasing the style. He argued that tension could be set up in the moment of release, similar to the electrophysiological trap mechanism in *Dionaea muscipula* [23].

Until now, it is not clear by which mechanism the style movement in Marantaceae is released. The **physiological release hypothesis** [12] proceeds from the view that membranes respond with depolarisation to the mechanical stimulus set by the pollinator. This would result in a decrease of turgor pressure on the upper side of the style allowing the cells at the lower side to expand and to separate from the hooded staminode. The specific histology of the style tissue supports this hypothesis [16].

The **mechanical release hypothesis** [12] on the contrary predicts that the movement of the trigger appendage would deform the hooded staminode thereby separating the style tip from the hood at the contact point [15]. The collapse of mechanical tension would affect the turgor change needed for the expansion of the lower cells. The lack of any sensory structures at the style surface opposite to the basal plate supports this hypothesis [16].

The present study aims to clarify the release process in the style movement of Marantaceae by experiment. For the first time, electrophysiological measurements are conducted to test how far a change of the membrane potential is involved and whether it is the cause or effect of the style movement.

## Material and Methods

### Plant material

*Goeppertia bachemiana* (E. Morren) Borchs. & S. Suárez and *Donax canniformis* (G. Forst.) K. Schum were used in the experiments. The species differ in their flower morphology. *Goeppertia bachemiana* has a stiff hooded staminode increasing mechanical pressure on the style, while *Donax canniformis* has a thin hooded staminode which appears to be not strong enough to hold the style under tension (Fig 2A–2D). Species names were confirmed according to the floras of Brazil and Thailand [24,25].

**Fig 3:**
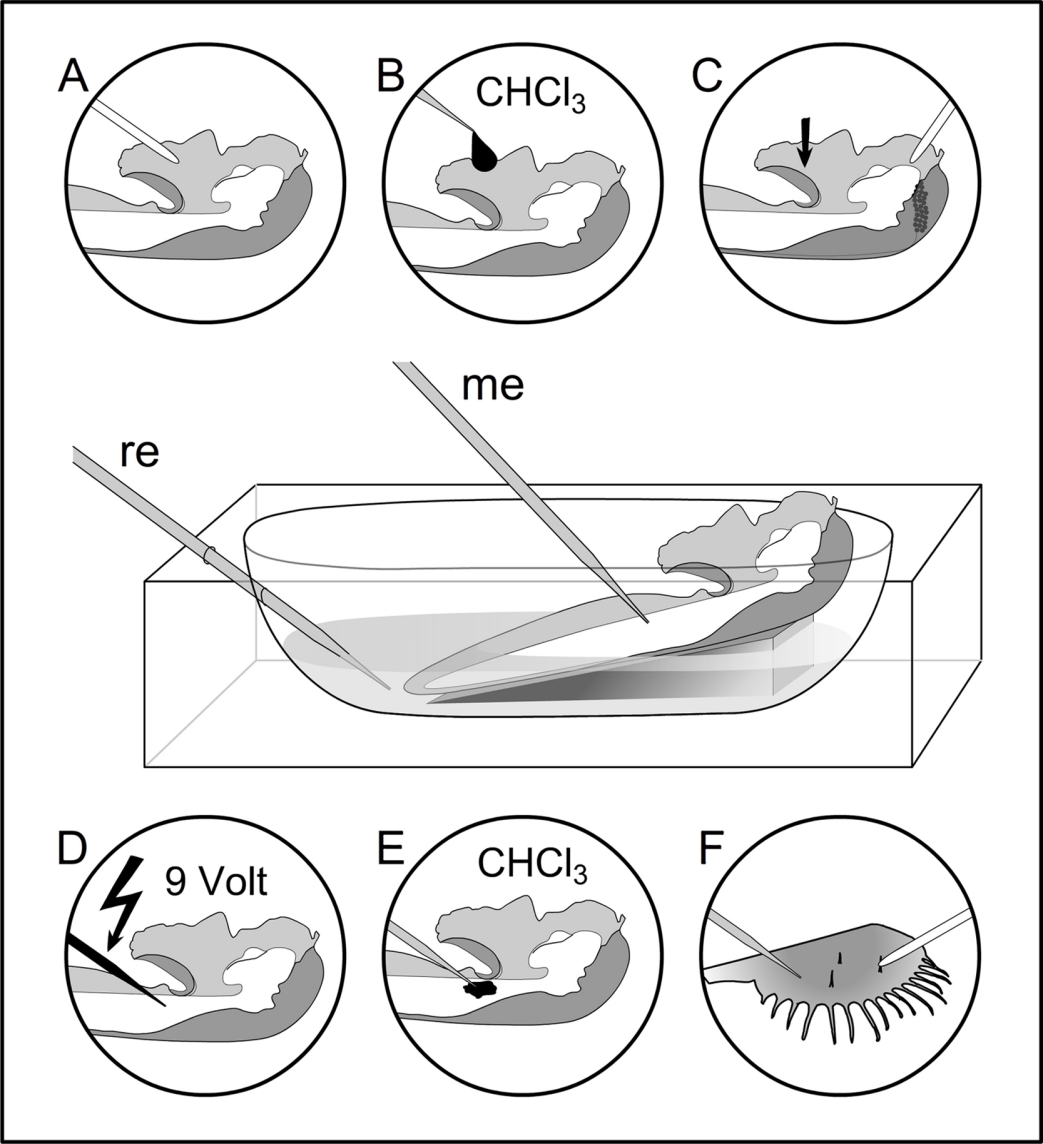
Experimental design for the extracellular measurements. The reference electrode (re) is positioned in an acrylic glass chamber through a small lateral hole. The fixed flowers were placed in water and the electrical change is measured using a microelectrode (me). (A) mechanical stimulation by deflecting the trigger appendage using a glass tube. (B) chemical stimulation by applying a droplet of chloroform with a syringe to the basal plate. (C) negative control experiment with an inhibited trigger appendage (basal plate is lifted, see arrow); the style is released by removing the hood with a class tube. (D) electrical stimulation, given via a minutien needle to test whether the style can be released electrically. (E) chemical stimulus by applying chloroform with a microelectrode to the style surface. (F) positive control; testing the electrophysiological equipment with a dissected leaf of *Dionaea muscipula*.

Fresh flowers were collected in the Botanical Gardens at Mainz (*Donax canniformis*) and Gießen (*Goeppertia bachemiana*). There is no specific permission required for these locations/activities since the material belongs to an old botanical collection. Corresponding to the ‘IUCN Red List’ both species are not endangered or protected. In view of the rapid wilting process, all flowers were picked in a fresh stage (the latest 6 h after the beginning of anthesis) and examined in the laboratory. Flowers were stored in a plastic box on wet tissue to keep them fresh (for max. 2 h). They were cut above the ovary and dissected to uncover style and hooded staminode. This functional unit was then fixed in a custom-made acrylic glass chamber (Fig 3) by using plasticine.

### Electrophysiological measurements

To test whether the release mechanism has an electrophysiological cause or not, extracellular measurements were conducted. Compared to intracellular measurements, this method is physically stable and less sensitive to vibrations [26].

**Fig 4:**
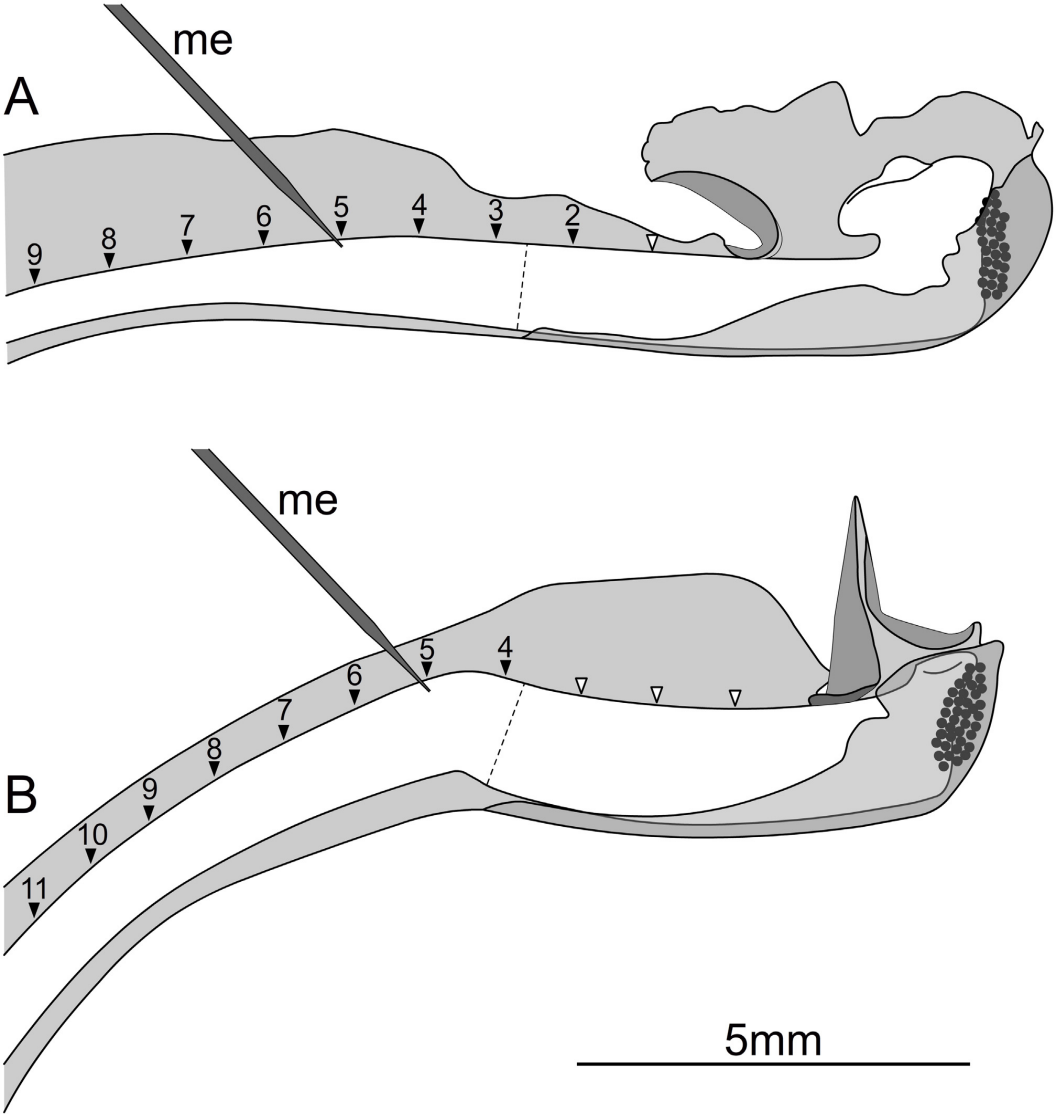
Positions for the extracellular measurements. Unreleased styles of *Donax canniformis* (A) and *Goeppertia bachemiana* (B). Measuring positions of the microelectrode (me) are indicated by black triangles that mark the distance (= D) to the basal plate in 1 mm steps. White triangles are omitted. The microelectrode is located at D = 5 mm in both schemata. Moving and non-moving part of the style is separated by a dotted line.

The fixed flowers were transferred to a faraday cage. Using a dissecting microscope and a screw micromanipulator, an apoplastic voltage electrode (Fig 3, me) was slightly inserted into the upper epidermal layer of the style to measure the extracellular voltage. To avoid a damage of the electrode due to the style movement, measurements were restricted to positions proximal to the bending tissue (Fig 4, black triangles). Measurements in the moving tissue of the style were only feasible in *Donax canniformis* that has a lower roll up angle of the style. The minimum distance (= D) between the electrode and the rim of the basal plate was 2 mm in *Donax canniformis* (Fig 4A) and 4 mm in *Goeppertia bachemiana* (Fig 4B). To close the circuit, the ovary side of the style was connected to the reference electrode (Fig 3, re) by covering both with tab water. Microelectrodes were made from borosilicate glass capillaries (OD = 1.5 mm, ID = 0.75 mm, with 0.2 mm filament; manufacturer: Hilgenberg) using a two-stage puller (L/M-3P-A, List-Medical) according to Felle and Zimmerman [27] and filled with 0.5 M KCl. To exclude intracellular recording the tip of the electrode was shortened up to 100 μm in diameter. For measurements a high-impedance (10^15^ Ω) Electrometer (FD223, World Precision Instruments, Sarasota, FL, USA) was used. Electric changes were traced on a chart recorder (W&W Recorder, Model 314) and analysed manually.

Each measurement started when the extracellular voltage showed a constant value for at least 120 s. This value was set as zero (0m V). Style movement was released by either deflecting the trigger appendage of the hooded staminode with a thin glass tube (Fig 3A) or by applying a droplet of chloroform to the basal plate (Fig 3B). Chloroform was chosen due to its physiological effect to membranes [14,28,29]. The corresponding electric response was recorded as extracellular voltage change (= ΔV).

To identify effects of conductivity, the measurements were repeated at different positions (Fig 4). Thereby the distance between electrode and proximal rim of the basal plate was extended in 1mm steps starting with the minimal distance (Fig 4). Due to different positions of the basal plate, measurements were conducted in a distance from 2 mm to 9 mm in *Donax canniformis* and from 4 mm to 11 mm in *Goeppertia bachemiana*. Each measurement and position was tested with a new flower (n = 488 for *G*. *bachemiana*, n = 94 for *Donax canniformis*).

### Inhibition of the trigger appendage

To test whether the style has a motor tissue opposite to the basal plate which would respond with electric changes when the trigger is moved [12], the latter was inhibited by anaesthetics. Then, the style was artificially released from the hooded staminode by removing the hood (Fig 3C). At the same time, electrophysiological measurements were conducted.

The anaesthetic manipulation was performed using chloroform steam [16,30]. For each species, the adequate concentration was tested to guarantee that the style movement would neither be blocked completely nor released automatically. Flowers of *Goeppertia bachemiana* were exposed to chloroform steam by dipping the flower for 1 s into a glass bottle (Schott BORO 100 ml; diameter: 60 mm) filled with 40ml chloroform. Flowers of *Donax canniformis* were placed in a petri dish for 2 min together with a small tank (diameter: 1.6 mm) filled with chloroform.

### Testing the influence of chloroform

Chloroform is well known for acting on membranes [29]. To test the hypothesis that the style movement can be released physiologically, experiments with chloroform droplets were conducted. As a reference, the Venus fly trap (*Dionaea muscipula*) was used, in which it was easily possible to close the leaf by a droplet of chloroform without touching the sensory hair. Similarly, in *Donax canniformis* and *Goeppertia bachemiana*, a droplet of chloroform was applied on the upper surface of the style, close to the trigger appendage. The droplet spread out and flow around the style. To avoid this effect, chloroform was applied with a microelectrode in a second experiment. For better visualization, the substance was stained with Sudan III. In each experiment, the extracellular potential was measured as described above.

### Testing the influence of electric impulses

To test style release by external electric signals, an experiment was conducted according to Volkov et al. [9]. Electric impulses (9 V battery) were given to the functional unit composed of style and hooded staminode with trigger appendage and basal plate (n = 10 flowers per species). For a better handling, a minutien needle was used as microelectrode and attached to the upper surface of the style (Fig 3D). To close the circuit, the battery was connected to the ovary side of the style. A switch was used to give electrical impulses (positive and negative) by hand. A weak connection or low conductivity of the electric circuit was excluded by testing the connection with a multimeter. This method was again successfully tested with the Venus flytrap (S1 Video).

### Video documentation

Representative experiments were recorded with a Canon G9 Digital Camera mounted with an adapter (Promicron) to the eyepiece of a dissecting microscope (Leica WILD M3C with flexible arm). A second camera (Sony DSC-TX1) was used at the same time to document the measurements of the chart recorder. By combining the two datasets, it is possible to visualise the measurements and results (online supplement).

### Statistical analysis

Extracellular voltage changes between different treatments and consecutive positions were tested with a two-sample t-test. The data was tested for a normally distribution before (Kolmogorov-Smirnov test) and in case of a deviation the Mann-Whitney-U-test was applied. All statistics were made with SPSS ver. 20.0.0.1.

## Results

*Dionaea muscipula*, used as a positive control, revealed that the equipment was suitable to measure extracellular electric changes.

### Mechanical release

**Fig 5:**
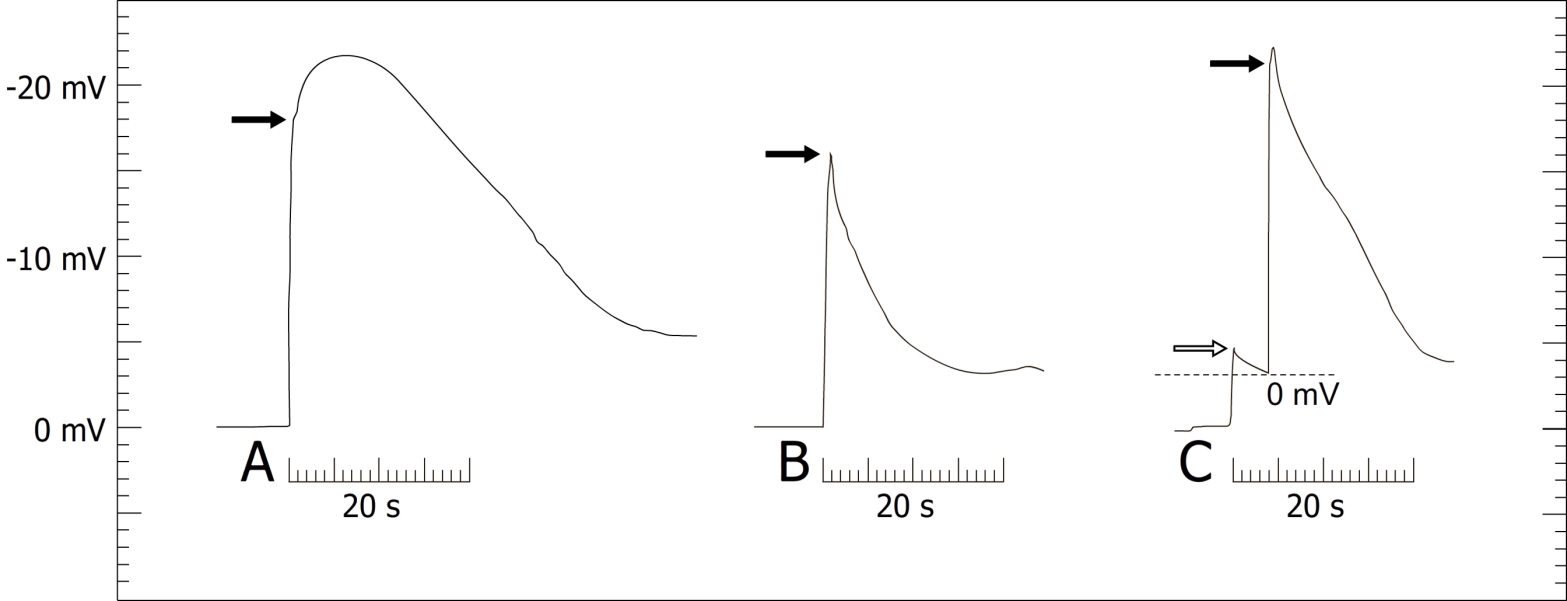
Amplitudes of the extracellular measurements. Style released by artificially deflecting the trigger appendage (A, B) or by applying a chloroform droplet (C) in *Donax canniformis* and *Goeppertia bachemiana*. The left border of the time bar (20 s) indicates the stimulus (t_o_); black arrows, apoplastic voltage change after 0.5 s; white arrow, pre-peak (only observed in chemical experiments). In C the style is released after the pre-peak drops, the corresponding ΔV-value was corrected (dotted line).

After the artificial deflection of the trigger appendage, the styles of both species show electric responses immediately (Fig 5, S1 Video). The apoplast usually hyperpolarized (Fig 5) except for few cases of depolarization. The amplitude was variable. There was either a distinct peak which directly declined indicating repolarization (Fig 5B) or a steep increase which was followed by a gradual rise and a delayed decline (Fig 5A, S1 Video). To nevertheless compare the data, the ΔV-values were always measured 0.5 s after style release.

**Fig 6:**
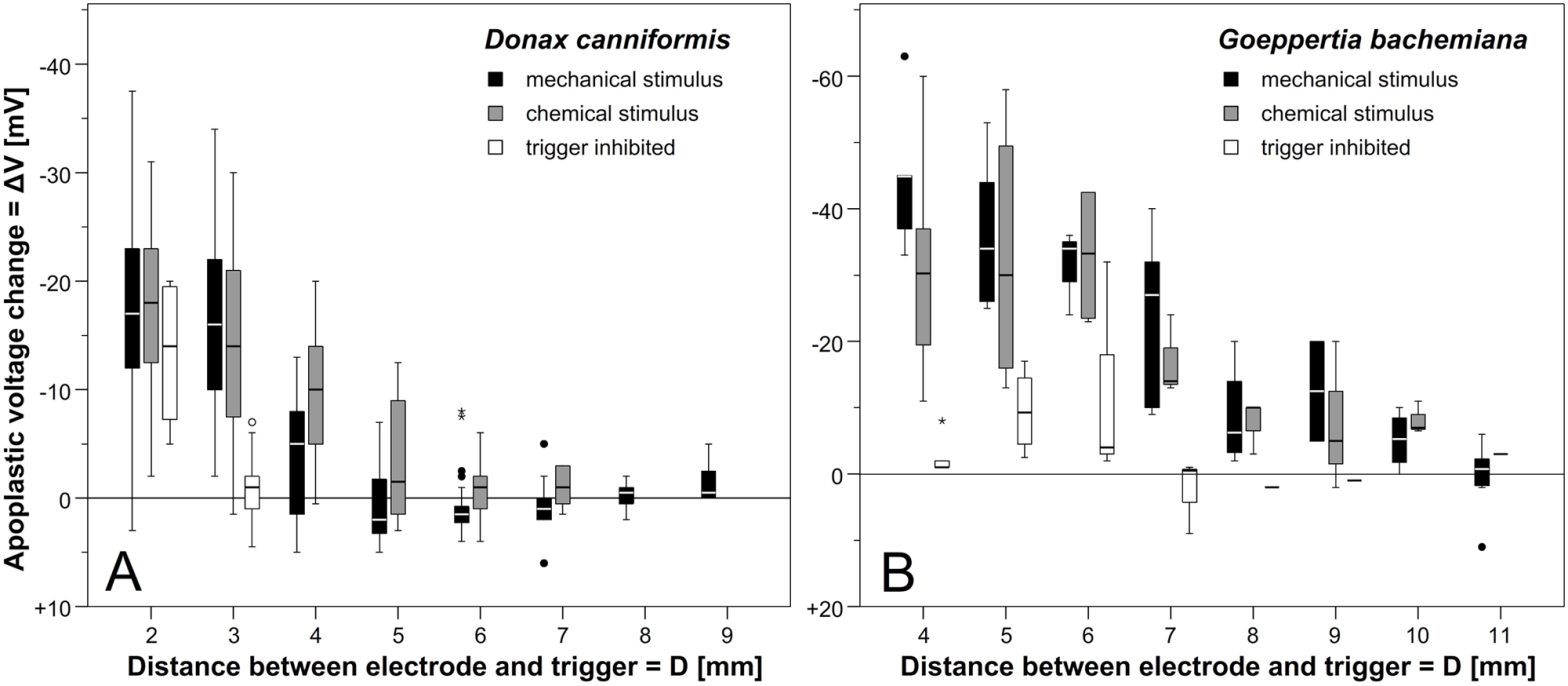
Apoplastic voltage change (ΔV) as a function of electrode position. (A) *Donax canniformis*, (B) *Goeppertia bachemiana*. Different treatments are indicated by colour: black, mechanical release, grey, chemical release, white, trigger inhibited (see S1 Table).

For both species, ΔV-values were analysed as a function of electrode position (Fig 6, S1 Table). In *Donax canniformis* (Fig 6A: black), the highest ΔV (most negative value) was measured in the distal-most position (D = 2 mm, mean: -17.14±1.22 mV SE; n = 57). From here, ΔV-values significantly decreased with distance (S2 Table) even reaching positive ΔV-values (up to +8 mV; Figs 4D and 5A) in a distance of 5–7 mm from the trigger appendage. The same pattern (decrease of measured ΔV as a function of position) was found in *Goeppertia bachemiana* (Fig 6B). However, hyperpolarization was generally higher in this species (D = 4mm, mean: -43.57±3.74 mV SE; n = 7) and depolarization less frequent than in *Donax canniformis* (S1 Table). Electric responses were only detected up to a distance of 9 mm in *D*. *canniformis* and to 11 mm in *Goeppertia bachemiana*.

The measurements clearly show that the mechanical release of the style movement goes along with an electric response which can be measured as a change in the extracellular voltage. This signal decreases with distance indicating that it is not self-perpetuating.

### Chloroform application

To clarify whether the voltage change is the cause or effect of the style release, experiments with chloroform were conducted. First, a chloroform droplet of undefined volume was applied on the upper surface of the style. In both species style movement was triggered by this treatment.

Style release either occurred at the very moment, the droplet touched the style or after a time lag of 1–10 s (S2 Video). In the first case, the amplitude was similar to the one measured after the mechanical style release (Fig 5A and 5B). In the second, chloroform induced a pre-peak (Fig 5C, S2 Video) followed by the main peak that appeared simultaneously with the style movement (Fig 5C). In both species the delayed cases were less frequent (*Donax canniformis*: 24.1% n = 45/187; *Goeppertia bachemiana*: 35.1% n = 13/37).

Similar to the mechanical treatments, ΔV-values decreased (less negative) in both species with distance (Fig 6, grey). However, chemical treatments show significant higher ΔV-values in certain positions e.g. in a distance of 4–7 mm in *Donax canniformis* (S1 Table).

During the experiments, it was observed that the chloroform flew under the basal plate into the hood of the staminode. Thereby, it was possible that the contact point between head and hood got separated (S3 Video). The same observation was made in previous studies with 70% alcohol (unpub. data). To exclude this kind of mechanical release, the experiment was repeated in a slightly different set up. A minute droplet of chloroform was applied to a very small limited area of the style surface. This treatment never released the style indicating that the results of the first experiment were indeed based on the mechanical separation of the head and hooded staminode. The second experiment clearly shows that the style movement cannot be triggered physiologically.

### Trigger inhibition

To clarify whether trigger deflection causes the electric response of the style or not, changes of the apoplastic (extracellular) voltage were measured after trigger inhibition (S4 Video). The style did not respond to trigger deflection and only curls up when the hood was removed. Simultaneously to the style movement an electric response was detected. The latter were generally lower than in the other treatments (Fig 6 white, S1 Table) indicating that the chloroform steam might influence the electric response. Measurements at the distal-most position (D = 2mm) in *Donax canniformis* were almost equal to mechanical and chemical treatments (S1 Table). This was the only position where the microelectrode was directly inserted into the moving tissue (Fig 4A). At a distance longer than 3 mm for *D*. *canniformis* and 9mm for *Goeppertia bachemiana*, no change of the apoplastic voltage was detectable any more.

### Electrical stimulation

Electric impulses did not release the style (S5 Video), neither by inserting the electrode deeper into the tissue nor by switching the voltage from positive to negative. Together with the chloroform experiments, it is thus evident, that the style release in Marantaceae differs from the electro-physiologically releasable turgor movement in the Venus fly trap.

## Discussion

Electrophysiological measurements clearly show that the style movements in *Donax canniformis* and *Goeppertia bachemiana* go along with an electric change in the apoplast. However, as it was not possible to stimulate style release electrophysiologically, the observed amplitudes (ΔV) are interpreted as the effect rather than the cause of the style movement.

### Variation potentials—a response to osmotic stress

Electric responses measured in Marantaceae clearly differ from action potentials documented for other plants, e.g. *Dionaea*. They show a slow and incomplete repolarization (not reaching the initial value) and lack self-perpetuation. Such amplitudes are known as slow wave or variation potentials (VPs). They are documented for *Mimosa* [26], tomatoes [31], cucumbers and pea seedlings [32]. The corresponding electric changes can be induced by chemicals, wounding, and heat [31] and are regarded as an effect of osmotic shocks [33] or hydraulic pressure waves [26]. Thus, VPs are electric responses of the tissue reacting on various kinds of stress. In Marantaceae, variation potentials are obviously caused by strong cell deformation (mechanical stress) going along with style movement. Histological investigations revealed that the style tissue has strongly perforated cell bundles. These were assumed to allow a fast water shift through the style tissue [16].

### Effects of chloroform

Electric measurements reveal that **chloroform droplets** produce a pre-peak in more than 20% of the measurements (*Donax canniformis*: 24.1%; *Goeppertia bachemiana*: 35.1%) indicating that they have an electrophysiological effect on the apoplast. However, this effect does not release the style as small chloroform droplets precisely applied at the upper side fail to initiate the movement (S3 Video). The style could only be released when large droplets flow into the hooded staminode. It is concluded that the close contact between style and hooded staminode (contact point) is released by this process resulting in a mechanical release of the style.

Experiments with **chloroform steam** indicate that the two species respond with different sensitivity to the narcotic agent [12,16]. This may be caused by their different proportions and shape, but still needs to be confirmed by comparative experiments. In both species, the trigger appendage and basal plate can be removed from the style after narcosis without releasing the style movement. Nevertheless, the style curls up in the usual way when the hood is removed afterwards. This experiment indicates that style tension is exclusively hold by the contact point and released mechanically. Obviously, there is no signal transfer between the basal plate and the style surface as assumed by several authors [12,14,22]. Thus, the existence of a motor tissue can be rejected.

Furthermore, styles treated with chloroform steam show similar electric responses as untreated styles (Fig 6A; white boxplot) only in the moving tissue. Obviously, the electric change is generated in the moving tissue and transmitted to the non-moving tissue in proximal direction. Compared to the ΔV-values measured in untreated flowers (Fig 6; black boxplot), the values in the non-moving tissue of narcotised flowers are significantly lower (Fig 6; white boxplot). The distinct curve progression implies that chloroform steam reduces the tissue conductivity, i.e. the signal transduction in the style. Since the molecular and cellular effect of chloroform is still unclear so far [28,29] further studies are needed to fully understand the effect of chloroform on moving tissues.

### Mechanical release of style movement

Rejecting the physiological release hypothesis implies that the explosive style movement in Marantaceae can be explained purely mechanically. In fact, the chloroform experiments revealed that style release is based on the ingress of the fluid in the space between style and hooded staminode thus separating the two organs mechanically (S3 Video). This ingress is promoted by the low surface tension of chloroform (26.67 mN/m) [34].

Based on his release experiments, Kunze (1984) argued in favour of an electrophysiological release mechanism. However, his results may be also explained by the mechanical model. When he removed part of the hood in two species of Marantaceae (*Maranta leuconeura* E. Morren, *Calathea undulata* Linden & André), he most likely did not release the pressure point [15]. When Kunze [22] and Claßen-Bockhoff and Heller [12] released the style by stinging under the basal plate it is likely that they lifted the basal plate and deformed the hooded staminode mechanically resulting in the separation of the style and hood at the contact point.

Such a deformation is also assumed to be the cause of the style release under natural conditions [12]. When a pollinator touches the trigger appendage a mechanical signal is transferred to the basal plate which due to its stiffness passes it on to the entire hooded staminode. The slight deformation of the hooded staminode causes a spatial change at the contact point and the release of style tension.

Pischtschan and Claßen-Bockhoff [14] argued that the turgor pressure in the upper side of the style would account for the storage of tension. This assumption is not in conflict with the present results. However, their preliminary conclusion that a change in the membrane potential would release the tension can be rejected by the data presented here. There are no hints for any electrophysiological release but instead good arguments supporting the mechanical release hypothesis.

## Conclusions

Experiments indicate that the explosive style mechanism in Marantaceae is triggered mechanically and that the electric responses are caused by the osmotic stress following the style movement. This finding reveals that fast movements in plants are not necessarily initiated by electric changes of the membrane as they are in the well-known example of *Dionaea*. Instead, complex mechano-physiological interactions between tissues and structures should be taken into consideration. Looking at a large taxonomic scale and comparing more genera with different morphologies might help to understand these interactions and the style mechanism in detail.

## Supporting Information

S1 Video Trigger deflection. Electric response after trigger deflection of *Donax canniformis*.
(MP4)

S2 Video Chloroform droplet. Electric response after applying a chloroform droplet to the style of *Goeppertia bachemiana*.
(MP4)

S3 Video Chloroform application. Chloroform precisely applied to the upper side of the style fails to initiate the movement in *Donax canniformis*.
(MP4)

S4 Video Trigger inhibition. Electric response of a narcotised flower of *Goeppertia bachemiana*. Since the trigger is inhibited the style can only be released by removing the hood.
(MP4)

S5 Video Electrical stimulation. Electric impulse applied to the midrib of the Venus flytrap (*Dionaea muscipula*) initiated the movement. The same treatment applied to style of *Donax canniformis* did not release the style.
(MP4)

S1 Table Statistical Tests between treatments. Two-sample t-tests or Mann-Whitney-U-test between treatments at different distances. P < 0.05 is bold.
(DOC)

S2 Table Statistical Tests between consecutive distances. Two-sample t-tests or Mann-Whitney-U-test between consecutive distances in mechanical, chemical and inhibited treatments. P < 0.05 is bold.
(DOC)
