# Supplementary material for: Electrical Signals in Prayer Plants (Marantaceae)? Insights into the Trigger Mechanism of the Explosive Style Movement
Source: PLoS One. 2015 May 21;10(5):e0126411. doi: 10.1371/journal.pone.0126411 (PMC4440630; doi:10.1371/journal.pone.0126411)
Supplement: S1 Table — Two-sample t-tests or Mann-Whitney-U-test between treatments at different distances. P < 0.05 is bold. (DOC) [file pone.0126411.s006.doc]

**Table S1**. **Statistical Tests between treatments.** Two-sample t-tests or Mann-Whitney-U-test between treatments at different distances. P < 0.05 is bold.

|  |  |  |  |  |  |  |  |  |
| --- | --- | --- | --- | --- | --- | --- | --- | --- |
| **Species** | **Distance** | **Treatment** | **N** | **mean ΔV [mV]** | **K-S test** | **tested groups** | **df** | **T-test or U-test** |
| *Donax* | 2 mm | mechanical | 57 | 17.140 ± 1.224 | Z = 0.738; P = 0.684 | mech. vs. chem. | 90 | T = 0.164; P = 0.870 |
| *canniformis* |  | chemical | 35 | 16.829 ± 1.384 | Z = 0.638; P = 0.810 | chem. vs. inhib. | 41 | T = -1.135; P = 0.263 |
|  |  | inhibited | 8 | 13.313 ± 2.254 | Z = 0.618; P = 0.839 | mech. vs. inhib. | 63 | T = 1.131; P = 0.262 |
|  | 3 mm | mechanical | 93 | 16.473 ± 0.811 | Z = 0.729; P = 0.663 | mech. vs. chem. | 177 | T = 2.256; P = 0.025 |
|  |  | chemical | 86 | 13.797 ± 0.867 | Z = 0.655; P = 0.785 | chem. vs. inhib. | 74.643 | T = -11.677; **P = 0.000** |
|  |  | inhibited | 18 | 0.806 ± 0.697 | Z = 0.583; P = 0.886 | mech. vs. inhib. | 70.414 | T = -14.651; **P = 0.000** |
|  | 4 mm | mechanical | 30 | 3.867 ± 1.002 | Z = 0.980; P = 0.292 | mech. vs. chem. | 54 | T = -3.353; **P = 0.001** |
|  |  | chemical | 26 | 9.154 ± 1.238 | Z = 0.485; P = 0.973 |  |  |  |
|  | 5 mm | mechanical | 31 | -0.968 ± 0.575 | Z = 0.997; P = 0.274 | mech. vs. chem. | 15.419 | T = -2.637; **P = 0.018** |
|  |  | chemical | 13 | 3.385 ± 1.547 | Z = 0.643; P = 0.803 |  |  |  |
|  | 6 mm | mechanical | 35 | -1.014 ± 0.443 | Z = 1.653; **P = 0.008** | mech. vs. chem. | 35/17 | U = 165; **P = 0.009** |
|  |  | chemical | 17 | 0.706 ± 0.650 | Z = 0.374; P = 0.999 |  |  |  |
|  | 7 mm | mechanical | 17 | -0.765 ± 0.541 | Z = 0.952; P = 0.325 | mech. vs. chem. | 24 | T = -2.136; **P = 0.043** |
|  |  | chemical | 9 | 1.111 ± 0.639 | Z = 0.847; P = 0.470 |  |  |  |
|  | 8 mm | mechanical | 5 | 0.200 ± 0.682 | Z = 0.398; P = 0.997 |  |  |  |
|  | 9 mm | mechanical | 8 | 1.375 ± 0.653 | Z = 0.769; P = 0.596 |  |  |  |
| *Goeppertia* | 4 mm | mechanical | 7 | 43.571 ± 3.747 | Z = 0.793; P = 0.555 | mech. vs. chem. | 21 | T = 2.103; **P = 0.048** |
| *bachemiana* |  | chemical | 16 | 30.906 ± 3.606 | Z = 0.557; P = 0.916 | chem. vs. inhib. | 18.202 | T = 7.343; **P = 0.000** |
|  |  | inhibited | 5 | 2.600 ± 1.364 | Z = 0.845; P = 0.473 | mech. vs. inhib. | 10 | T = 8.837; **P = 0.000** |
|  | 5 mm | mechanical | 5 | 36.400 ± 5.372 | Z = 0.462; P = 0.983 | mech. vs. chem. | 7 | T = 0.334; P = 0.748 |
|  |  | chemical | 4 | 32.750 ± 10.347 | Z = 0.494; P = 0.968 | chem. vs. inhib. | 3.558 | T = 2.149; P = 0.107 |
|  |  | inhibited | 4 | 9.500 ± 3.169 | Z = 0.364; P = 0.999 | mech. vs. inhib. | 7 | T = 4.017; **P = 0.005** |
|  | 6 mm | mechanical | 3 | 31.333 ± 3.712 | Z = 0.567; P = 0.904 | mech. vs. chem. | 4.850 | T = -0.252; P = 0.812 |
|  |  | chemical | 4 | 33.000 ± 5.489 | Z = 0.613; P = 0.846 | chem. vs. inhib. | 6 | T = 2.489; **P = 0.047** |
|  |  | inhibited | 4 | 10.500 ± 7.182 | Z = 0.849; P = 0.467 | mech. vs. inhib. | 5 | T = 2.303; P = 0.070 |
|  | 7 mm | mechanical | 6 | 24.167 ± 5.056 | Z = 0.507; P = 0.959 | mech. vs. chem. | 7 | T = 0.925; P = 0.386 |
|  |  | chemical | 3 | 17.000 ± 3.519 | Z = 0.616; P = 0.842 | chem. vs. inhib. | 4 | T = 4.073; **P = 0.015** |
|  |  | inhibited | 3 | -2.500 ± 3.253 | Z = 0.640; P = 0.807 | mech. vs. inhib. | 7 | T = 3.463; **P = 0.011** |
|  | 8 mm | mechanical | 4 | 8.625 ± 3.986 | Z = 0.562; P = 0.910 | mech. vs. chem. | 5 | T = 0.188; P = 0.858 |
|  |  | chemical | 3 | 7.667 ± 2.333 | Z = 0.667; P = 0.766 | chem. vs. inhib. | 2 | T = 2.071; P = 0.174 |
|  |  | inhibited | 1 | -2 |  | mech. vs. inhib. | 2 | T = -2.071; P = 0.174 |
|  | 9 mm | mechanical | 2 | 12.500 ± 7.500 | Z = 0.368; P = 0.999 | mech. vs. chem. | 3 | T = 0.480; P = 0.664 |
|  |  | chemical | 3 | 7.667 ± 6.489 | Z = 0.451; P = 0.987 | chem. vs. inhib. | 2 | T = -0.668; P = 0.573 |
|  |  | inhibited | 1 | -1 |  | mech. vs. inhib. | 1 | T = -1.039; P = 0.488 |
|  | 10 mm | mechanical | 4 | 5.125 ± 2.164 | Z = 0.335; P = 1.000 | mech. vs. chem. | 5 | T = 1.077; P = 0.331 |
|  |  | chemical | 3 | 8.167±1.424 | Z = 0.604; P = 0.859 |  |  |  |
|  | 11 mm | mechanical | 8 | -0.313 ± 1.773 | Z = 0.688; P = 0.731 | mech. vs. chem. | 7 | T = 0.623; P = 0.553 |
|  |  | chemical | 1 | 3 |  |  |  |  |
