# Supplementary material for: Electrical Signals in Prayer Plants (Marantaceae)? Insights into the Trigger Mechanism of the Explosive Style Movement
Source: PLoS One. 2015 May 21;10(5):e0126411. doi: 10.1371/journal.pone.0126411 (PMC4440630; doi:10.1371/journal.pone.0126411)
Supplement: S2 Table — Two-sample t-tests or Mann-Whitney-U-test between consecutive distances in mechanical, chemical and inhibited treatments. P < 0.05 is bold. (DOC) [file pone.0126411.s007.doc]

**Table S2. Statistical Tests between consecutive distances.** Two-sample t-tests or Mann-Whitney-U-test between consecutive distances in mechanical, chemical and inhibited treatments. P < 0.05 is bold.

|  |  |  |  |  |
| --- | --- | --- | --- | --- |
| **Species** | **Treatment** | **tested Distances** | **df** | **T-test or U-test** |
| *Donax* | mechanical | 2 mm vs. 3 mm | 148 | T = 0.473; P = 0.637 |
| *canniformis* |  | 3 mm vs. 4 mm | 69.976 | T = -9.777; **P = 0.000** |
|  |  | 4 mm vs. 5 mm | 46.351 | T = 4,185; **P = 0.000** |
|  |  | 5 mm vs. 6 mm | 58.157 | U = 496.5; P = 0.553 |
|  |  | 6 mm vs. 7 mm | 50 | U = 229.5; P = 0.178 |
|  |  | 7 mm vs. 8 mm | 20 | T = 0.900; P = 0.379 |
|  |  | 8 mm vs. 9 mm | 11 | T = -1.187; P = 0.260 |
|  | chemical | 2 mm vs. 3 mm | 119 | T = 1.871; P = 0.064 |
|  |  | 3 mm vs. 4 mm | 110 | T = -2.700; **P = 0.008** |
|  |  | 4 mm vs. 5 mm | 37 | T = 2,792; **P = 0.008** |
|  |  | 5 mm vs. 6 mm | 16.233 | T = -1,740; P = 0.093 |
|  |  | 6 mm vs. 7 mm | 24 | T = -0.401; P = 0.692 |
|  | inhibited | 2 mm vs. 3 mm | 8.371 | T = 5.302; P = 0.000 |
| *Goeppertia* | mechanical | 4 mm vs. 5 mm | 10 | T = 1.134; P = 0.283 |
| *bachemiana* |  | 5 mm vs. 6 mm | 6 | T = -0.662; P = 0.533 |
|  |  | 6 mm vs. 7 mm | 7 | T = 0.920; P = 0.388 |
|  |  | 7 mm vs. 8 mm | 8 | T = -2.201; P = 0.059 |
|  |  | 8 mm vs. 9 mm | 4 | T = -0.514; P = 0.634 |
|  |  | 9 mm vs. 10 mm | 4 | T = -1.311; P = 0.260 |
|  |  | 10 mm vs. 11 mm | 10 | T = 1.843; P = 0.095 |
|  | chemical | 4 mm vs. 5 mm | 18 | T = -0.211; P = 0.835 |
|  |  | 5 mm vs. 6 mm | 6 | T = 0.021; P = 0.984 |
|  |  | 6 mm vs. 7 mm | 5 | T = 2.245; P = 0.075 |
|  |  | 7 mm vs. 8 mm | 4 | T = -2.214; P = 0.091 |
|  |  | 8 mm vs. 9 mm | 4 | T = 0.000; P = 1.000 |
|  |  | 9 mm vs. 10 mm | 4 | T = 0.075; P = 0.944 |
|  |  | 10 mm vs. 11 mm | 2 | T = 1.814; P = 0.211 |
|  | inhibited | 4 mm vs. 5 mm | 7 | T = -2.167; P = 0.067 |
|  |  | 5 mm vs. 6 mm | 6 | T = 0.127; P = 0.903 |
|  |  | 6 mm vs. 7 mm | 5 | T = 1.457; P = 0.205 |
|  |  | 7 mm vs. 8 mm | 2 | T = 0.077; P = 0.946 |
|  |  | 8 mm vs. 9 mm | 0 |  |
